# Multimodal imaging of a case of peripheral cone dystrophy

**DOI:** 10.1007/s10633-015-9490-1

**Published:** 2015-02-24

**Authors:** Naoko Ito, Shuhei Kameya, Kiyoko Gocho, Takaaki Hayashi, Sachiko Kikuchi, Satoshi Katagiri, Tamaki Gekka, Kunihiko Yamaki, Hiroshi Takahashi, Hiroshi Tsuneoka

**Affiliations:** 1 Department of Ophthalmology, The Jikei University School of Medicine, 3-25-8 Nishi-shimbashi, Minato-ku, Tokyo 105-8461 Japan; 2 Department of Ophthalmology, Nippon Medical School Chiba Hokusoh Hospital, 1715 Kamagari, Inzai, Chiba 270-1694 Japan; 3 Department of Ophthalmology, Nippon Medical School, 1-1-5 Sendagi, Bunkyo-ku, Tokyo 113-8602 Japan

**Keywords:** Peripheral cone dystrophy, Adaptive optics, Parafovea, Cone density

## Abstract

**Purpose:**

To characterize the peripheral cones in the images obtained by spectral-domain optical coherence tomography (OCT), swept source OCT, and adaptive optics fundus camera in a patient with peripheral cone dystrophy.

**Methods:**

A 28-year-old Japanese man underwent detailed ophthalmic evaluations including high-resolution imaging of the fundus of both eyes.

**Results:**

The decimal best-corrected visual acuity was 1.2 in both eyes. The results of slit-lamp biomicroscopy and ophthalmoscopy were essentially normal. Fluorescein and indocyanine green angiographies did not show any hyper- or hypofluorescent areas of the retina. Goldmann perimetry showed full peripheral visual fields but relative central scotomas within the central 20°. The results of the Humphrey Visual Field Analyzer showed a limited preservation of the central sensitivity. Color vision tests showed no errors in both eyes. Spectral-domain OCT showed attenuation of both the ellipsoid and interdigitation zones throughout the macular region except the center of the fovea. The scotopic full-field ERGs were normal, but the photopic ERGs were markedly reduced. Regular cone mosaics were not observed especially more than 450 μm radius from the fovea in the adaptive optics retinal images. The parafoveal cone densities were severely decreased in both eyes.

**Conclusions:**

Our findings indicate that the peripheral cone dystrophy diagnosed by full-field ERGs and perimetry is due to a reduction in the density of parafoveal and peripheral cones.

## Introduction

Cone dystrophy is a type of hereditary retinal degeneration characterized by a progressive dysfunctioning of the cone photoreceptors. The possibility of cone dystrophy confined mainly in the periphery had been suggested [1, 2], and Krill et al. reported three patients with cone dystrophy, who had normal or near-normal visual acuities and normal color vision but had peripheral cone dysfunction [2]. Peripheral cone dystrophy (PCD) is a very rare clinical entity, and its clinical characteristics have been presented in only six reports [2–7]. Kondo et al. [3] described the clinical features of three patients with PCD in which the peripheral cone system was more affected than the central cone system, and the rod system was completely preserved. Okuno et al. reported on an elderly male patient with PCD whose symptoms had not changed for over 50 years. They concluded that the signs and symptoms of these patients were not manifested at an early stage in the more common type of PCD [4]. Mochizuki et al. [5] presented a 34-year-old patient with unilateral peripheral cone dysfunction.

The adaptive optics (AO) fundus camera can obtain images with a transverse resolution of <2 µm which makes it possible to resolve individual cone photoreceptors and other retinal structures in living human eyes [8–10]. This technique has been used to analyze the cone photoreceptor mosaic in eyes with various inherited retinal degenerations [9, 11–13]. An increase in the cone spacing, i.e., a reduction of cone density, in retinas with cone–rod dystrophy has been detected by AO imaging [9, 11, 12]. The degree of reduction of the cone density was correlated with the decrease in visual function measured by multifocal electroretinography (mfERG) [9, 11, 12]. A dark area in the AO fundus images was reported to be caused by disruptions of the interdigitation zone (IZ; formerly called the cone outer segment tips line) in the spectral-domain optical coherence tomographic (SD-OCT) images [14, 15].

We report our findings in a PCD patient who was examined by high-resolution imaging of the central and peripheral cone photoreceptors with high-resolution OCT and AO analyses.

## Methods

The protocol of this study conformed to the tenets of the Declaration of Helsinki and was approved by the Institutional Review Board of the Nippon Medical School. A written informed consent was signed by all participants after an explanation of the purpose of this study and possible complications.

A 28-year-old Japanese man (JU#0751) was diagnosed with PCD by the findings of full-field ERGs and perimetry. He underwent detailed ophthalmic evaluations.

### Clinical studies

The ophthalmological examinations included measurements of the best-corrected visual acuity (BCVA), slit-lamp biomicroscopy, and dilated funduscopy. Short-wavelength autofluorescence (488 nm) and fluorescein and indocyanine green angiography (FA and ICGA) were performed with a confocal scanning laser ophthalmoscope (Spectralis HRA; Heidelberg Engineering, Heidelberg, Germany). The visual fields were obtained by Goldmann kinetic perimetry (Haag Streit, Bern, Switzerland) and Humphrey Visual Field Analyzer (Carl Zeiss Meditec, Inc, Dublin, CA, USA). The Swedish interactive threshold algorithm standard strategy was used with program 30-2 of the Humphrey Visual Field Analyzer.

### Electroretinograms (ERGs)

Full-field ERG was recorded by Burian–Allen corneal contact lens electrodes with an EOG-ERG Ganzfeld stimulator (Electrophysiology system; LACE Elettronica, Pisa, Italy) according to the recommendation of the International Society for Clinical Electrophysiology of Vision (ISCEV) [16].

The mfERGs were recorded with an mfERG stimulating and recording system (VERIS Science; Electro-Diagnostic Imaging, Inc. Redwood City, CA, USA) [17, 18]. The mean luminance of the stimulus was 103 cd/m^2^, and the contrast was 95 %. The overall stimulus area subtended approximately 40° at the cornea, and the frame rate was 75 Hz. The pseudorandom stimulus presentation, the m-sequence, was at 2^14^–1, and each run was divided into eight equal segments with a total recording time of about 4 min.

### High-resolution imaging analyses

The SD-OCT images were acquired with a Cirrus HD-OCT (Carl Zeiss Meditec). The B-scan retinal images were composed of 27,000/s consecutive A-scans passing through the center of the macula horizontally. In total, 1024 A-scans are contained in a B-scan image and 20 images are averaged. The fixation was centered on the fovea. The total scan depth was 2 mm, the axial resolution was 5 μm, and transverse resolution was 15 μm. Since the single line scan SD-OCT images are 6 mm long, an actual size was calculated by their pixel ratio. The 512 × 128 macular cube scan protocol was used to obtain the retinal thickness map. With this protocol, 128 cross-sectional B-scan images were obtained with each composed of 512 A-scans.

*En face* OCT images were obtained by swept source optical coherence tomography (DRI OCT-1 Atlantis, Topcon, Japan). The fixation was centered on the fovea. After the acquisition of cross-sectional B-scan images, *en face* OCT images were reconstructed by the *EN*-*VIEW* program (Topcon, Japan). The flattening of the 3D-OCT images based on the retinal-pigmented epithelium layer was performed, and then, a scan line was adjusted to IZ zone to acquire *en face* images. High-resolution fundus images were taken using the flood-illuminated AO retinal camera (rtx1, Imagine Eyes, Orsay, France). This system has been utilized to image individual cone photoreceptors [13, 19–21] and other retinal structures [10, 21, 22]. The AO fundus camera illuminated a 4° square field with 850-nm infrared flashes to acquire *en face* images of the retina with a transverse optical resolution of 250 line pairs/mm. Successive AO images were taken at adjacent retinal locations with an angular spacing of 2° in the horizontal and vertical directions. This procedure allowed an overlap of the horizontal and vertical images of at least 2°. Prior to each acquisition, the focus depth was adjusted to the region corresponding to the ellipsoid zone (EZ: also termed inner segment/outer segment junction) and the IZ in the SD-OCT images. The resulting images were stitched together by superimposing retinal vessel landmarks by an image editing software (GIMP, The GIMP Development Team; Image J, National Institute of Health, Bethesda, MD). The size of each pixel was typically 0.8 µm when calculated at the retinal plane, and the values were adjusted for variations in the axial length of the eye [23]. To evaluate the cone patterns of normal controls and the patient with PCD, we used the automated cone labeling analysis software (AOdetect; Imagine Eyes). AOdetect was developed by Imagine Eyes and allows researchers to obtain both the local density and a mean density within a user-defined region of interest. AOdetect also includes the program to calculate an actual length in the images by entering the axial length of the eye. The positions of the cone photoreceptors were computed by automatically detecting the central coordinates of small circular spots where the brightness was higher than the surrounding background. First, the averaged image without contrast adjustments was filtered to locate the maxima of the image. The spatial distribution of these points was analyzed using Voronoi diagrams where the detected points served as generators. After automated cone labeling, the estimated cone labeling was manually verified by three investigators to minimize any potential cone under- or oversampling made by the automated software. As has been reported for similar systems, we found that our system did not always have a clear view of the individual cones within much of the central area. However, we could clearly distinguish individual cones at sites >450 µm from the fovea. Therefore, we obtained an estimate of cone density in a 50 × 50 µm area at 600 µm from the foveal center.

The automated cone labeling did not identify each cone precisely in the images taken from the region with severe photoreceptor degeneration. To estimate the cone density of the patient, we manually selected circular spots of more than 4 µm in the images where the brightness was obviously higher than the surrounding background level. The density of the cones was measured by three investigators separately to minimize any potential under- or oversampling of the cones. We examined the cone density at 600 μm nasal from the fovea and also the axial length of 16 normal control eyes. There were 11 men and 5 women whose age ranged from 22 to 45 years (mean, 33.9 ± 8.0 years) in this control group. We calculated the 95 % confidence intervals, 95 % prediction intervals, and *R* ^2^ value of regression line of the cone density of normal controls. We also calculated mean and standard deviation of cone density of normal controls.

## Results

### Case report

A 28-year-old man (JU#0751) first visited the Department of Ophthalmology of The Jikei University School of Medicine because of photophobia and difficulty in following objects in motion. He has had these symptoms for over 5 years, and they had become progressively worse. His non-consanguineous parents were unaffected, and he had no significant medical history. Patient has no history of hydroxychloroquine, chloroquine, nor other retinotoxic drug use. Magnetic resonance (MR) imaging and MR angiography of the brain showed no abnormalities.

**Fig. 1:**
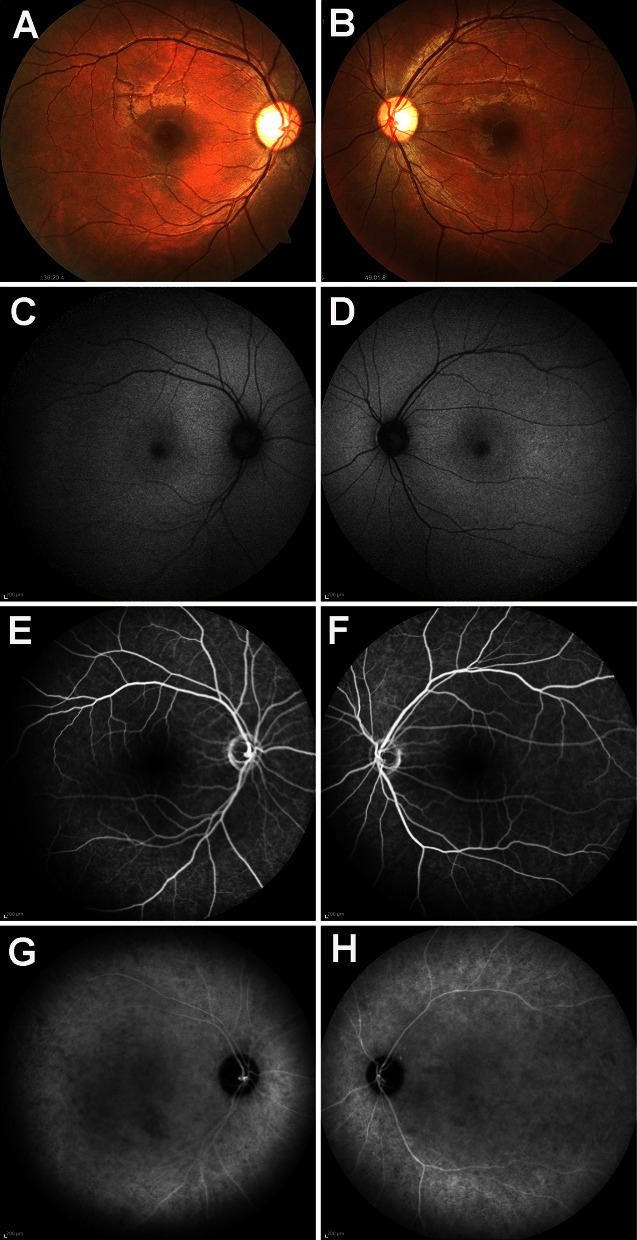
Fundus photographs, short-wavelength autofluorescence image, and fluorescein and indocyanine green angiograms from a patient with peripheral cone dystrophy (PCD). Fundus photographs (**a**, **b**), short-wavelength autofluorescence (**c**, **d**), fluorescein angiograms (**e**, **f**), and indocyanine green angiograms (**g**, **h**) are shown. Results from the right eye (**a**, **c**, **e**, **g**) and left eye (**b**, **d**, **f**, **h**) are shown. Fundus photographs show no abnormal findings in both maculas. Mild temporal pallor of the optic disks can be seen. Short-wavelength autofluorescence showed a subtle parafoveal hyperfluorescence. Mid-phase fluorescein and indocyanine green angiograms do not show any hyper- or hypofluorescent regions

On the initial evaluation, his decimal BCVA was 1.2 with mild myopia of −2.75 diopters in both eyes. The results of slit-lamp biomicroscopy were normal, and ophthalmoscopy showed mild temporal pallor of the optic disk but both maculas were normal (Fig. 1a, b). Short-wavelength autofluorescence showed a subtle parafoveal hyperfluorescence (Fig. 1c, d). FA and ICGA did not show any hyper- or hypofluorescent regions at any phase (Fig. 1e–h).

**Fig. 2:**
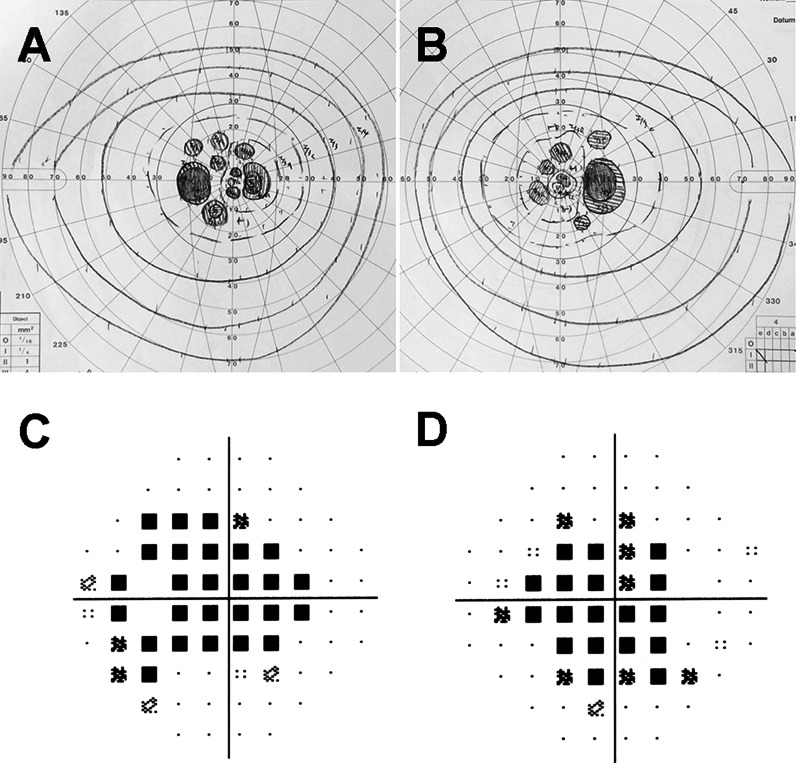
Visual fields of PCD patient. Results of Goldman kinetic perimetry (**a**, **b**) and pattern standard deviation of Humphrey Visual Field Analyzer (**c**, **d**) are shown. Results from the right eye (**b**, **d**) and left eye (**a**, **c**) are shown. Goldmann visual field tests show that the peripheral visual fields are full, but a relative central scotoma is present within 20° of the fovea in both eyes. Humphrey visual field testing (30-2) shows a relative central scotoma within 20° in right eye and 30° in left eye in the PCD patient. The central sensitivity is preserved locally in both eyes

Goldmann visual field tests showed that the peripheral visual fields were full, but a relative scotoma was found within 20° of the fovea in both eyes (Fig. 2a, b). The results of Humphrey Visual Field Analyzer with the central 30-2 SITA-standard program showed mean deviation (MD) values of −9.98 dB for the right eyes (*P* < 0.5 %) and −9.78 dB for the left eyes (*P* < 0.5 %). The pattern standard deviation (PSD) values were 5.21 dB for the right eye (*P* < 0.5 %) and 7.53 dB for the left eye (*P* < 0.5; Fig. 2c, d). The central sensitivity was not altered significantly in both eyes.

The color vision assessed monocularly with the Ishihara test (38-plate edition) and the Farnsworth Panel D-15 (Panel D-15) was normal in both eyes.

**Fig. 3:**
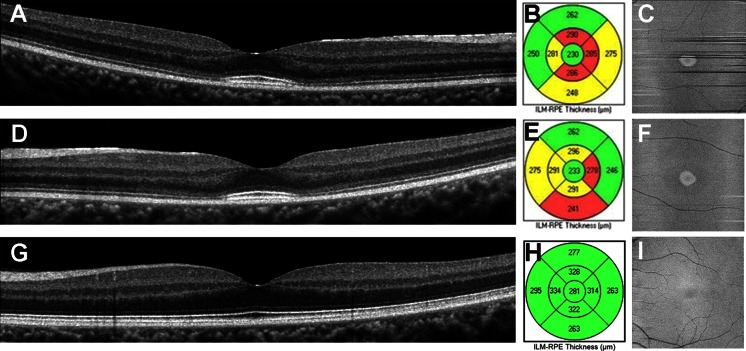
Spectral-domain optical coherence tomographic (SD-OCT) images. Images of horizontal SD-OCT scan (**a**, **d**, **g**), macular thickness maps (**b**, **e**, **h**), and *en face* SD-OCT are shown (**c**, **f**, **i**). Images from the right eye (**a**–**c**) and left eye (**d**–**f**) of a patient with PCD and left eye of normal control (**g**–**i**) are shown. SD-OCT shows attenuation of both the EZ and IZ throughout the region except for about 1 mm at the center of the fovea. The retinal thickness map shows normal macular thickness within 1 mm diameter of the foveal center, but the parafoveal areas are thinner. Normal control showed clearly distinguishable EZ and IZ throughout the entire region. *En face* OCT images of the patient show an oval, high-intensity area surrounded by low-intensity region. Note that the OCT findings in both eyes of the patient are similar

The SD-OCT images showed a disruption of both the EZ and IZ throughout the macular region except at the foveal center (Fig. 3a, d). The macular thickness was normal only within 1 mm diameter of the foveal center, whereas the paracentral areas were thinner (Fig. 3b, e). The IZ reflectance in the central fovea is slightly wider and brighter than normal.

*En face* OCT images with a scan line adjusted to the level of the IZ showed an oval, high-intensity area that corresponded to residual IZs in the B-scan OCT images (Fig. 3c, f). The high-intensity area was surrounded by a low-intensity area corresponding to an attenuation of the IZ in the B-scan OCT images (Fig. 3c, f). Similar OCT findings were observed in both eyes.

**Fig. 4:**
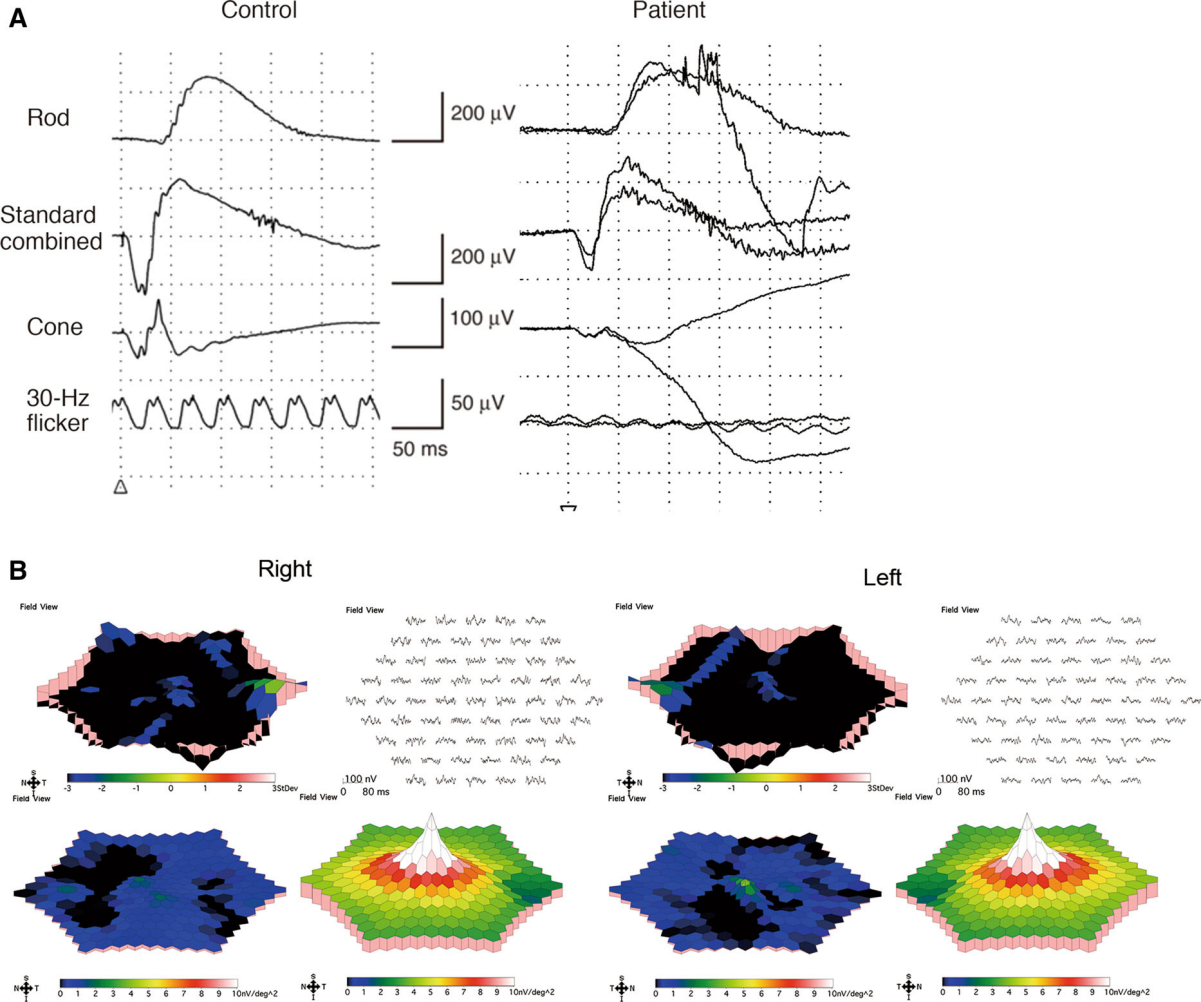
Full-field and multifocal electroretinograms (ERGs). Full-field and multifocal ERGs recorded from normal control and the PCD patient are shown. The dark-adapted 0.01, dark-adapted 3.0, light-adapted 3.0, and light-adapted 3.0 flicker ERGs of full-field ERGs are shown (**a**). The amplitudes and implicit times of the dark-adapted 0.01 and dark-adapted 3.0 are normal. The amplitudes of the b-wave of the light-adapted 3.0 and light-adapted 3.0 flicker responses are markedly reduced in both eyes. The results of mfERGs showed slightly detectable responses in the central area of left eye; however, the amplitudes of the other area were severely reduced in the patients (**b**)

The amplitudes and implicit times of the scotopic full-field ERGs elicited by 0.01 and 3.0 stimuli were normal (Fig. 4a). The amplitudes of the b-waves of the photopic ERGs elicited by 3.0 stimuli and the photopic light-adapted 3.0 flicker responses were markedly reduced in both eyes (Fig. 4a). The results of mfERGs showed slightly detectable responses in the central area of left eye; however, the amplitudes of the other area were severely reduced in the patients (Fig. 4b).

### High-resolution imaging analyses

**Fig. 5:**
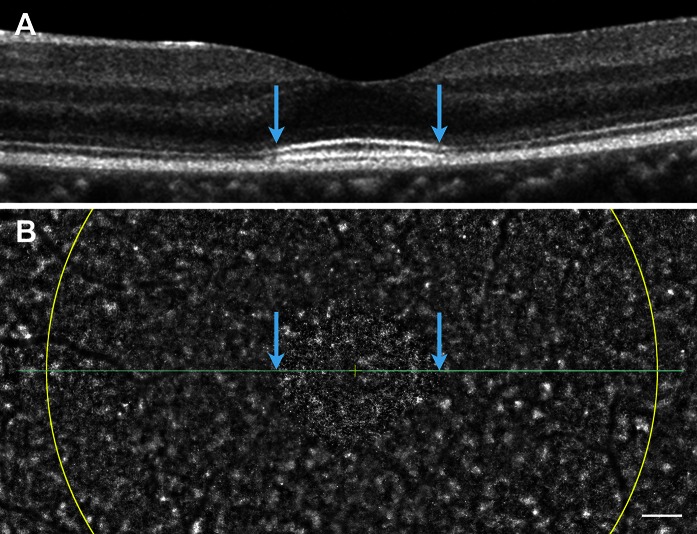
SD-OCT and low-magnification AO images. SD-OCT (**a**) and low-magnification AO (**b**) images are shown. The width of the SD-OCT image in **a** corresponds to the width of the AO image in **b**. *Blue arrows* in **a** indicate the edge of discernable EZ and IZ. *Blue arrows* in **b** point to the same location in **a**. *Yellow cross* in **b** indicates the fixation point. *Yellow line* in **b** indicates 5° circle from the fixation point. Note that cone density of areas more than 450 μm from the fovea is very low. The oval area of residual cone photoreceptors in **b** was similar to the shape of residual IZ in the *en face* OCT image (Fig. 3f). *Bar* 200 μm

**Fig. 6:**
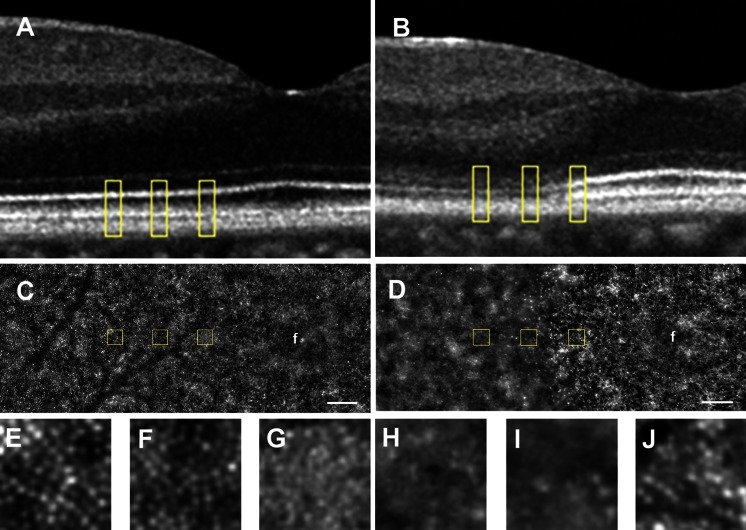
SD-OCT and high-magnification AO images. Magnified SD-OCT images of a normal control (**a**) and PCD patient (**b**) are shown. *Yellow boxes* in **a** and **b** are nasal areas at 600, 450, and 300 μm away from the foveal center. A montage of low-magnification AO image of normal control (**c**) and the patient (**d**) is shown. *Yellow boxes* in **c** and **d** are also nasal areas at 600, 450, and 300 μm away from the foveal center (**f**). (**e**–**j**) Magnified view of the area outlined in **c** and **d** is shown. The area at 600 μm (**e**), 450 μm (**f**), and 300 μm (**g**) nasal from the foveal center in normal control is shown. The area at 600 μm (**h**), 450 μm (**i**), and 300 μm (**j**) nasal from the foveal center in the patient is shown. Regular cone mosaics are not observed especially more than 450 μm from the fovea in the patient, while AO image of normal control shows well-ordered cone mosaic. *Bar* 100 μm

High-resolution *en face* AO imaging did not detect regular cone mosaics especially more than 450 μm from the fovea, while the AO images of normal control showed well-ordered cone mosaic in this area (Figs. 5b, 6c–j). Similar results were observed in both eyes. The oval area of residual cone photoreceptors in the AO image was similar to the shape of residual IZ in the *en face* OCT image (Fig. 3f and between blue arrows in Fig. 5b).

**Fig. 7:**
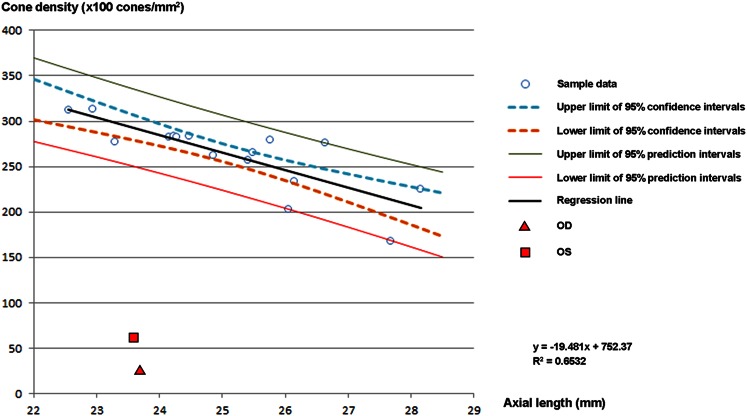
Statistical data of adaptive optics analyses. Relationship between cone density and axial length was obtained from 16 normal control samples. There was a statistically significant negative correlation between cone density and axial length (*R* ^2^ = 0.6532). Upper and lower limits of 95 % confidence intervals, 95 % prediction intervals, and regression line of normal controls are shown. The results of estimated cone density and axial length of the patient are plotted as indicated *marks*

We also examined the cone density at 600 μm nasal from the fovea and the axial length of 16 normal control eyes. The values were compared to the findings in the cone density at 600 μm nasal from the fovea in the patient (Fig. 7). It is well known that there is a significant negative correlation between the axial length and cone density [24, 25], and the correlation coefficient was 0.6532 for the normal controls. The mean and standard deviation of the cone density of this normal control group were 262.8 ± 38.5 (×100 cones/mm^2^). The cone densities of both eyes in the patient were severely decreased and well outside the standard deviation and 95 % prediction interval of the normal controls (Fig. 7).

## Discussion

The concept of regional cone dystrophy has been proposed by some clinicians, and eyes with regional cone dystrophy were thought to have either a central or peripheral cone dystrophy [1, 2, 26]. In eyes diagnosed with PCD, the cone system is impaired predominantly in the periphery, and the rod system is completely preserved even in areas where the cone system is impaired [1, 2]. The results of an earlier study showed that patients with PCD retained normal or near-normal visual acuities and normal color vision [1]. Our patient had normal visual acuity, normal color vision, and normal scotopic full-field ERGs in both eyes in spite of a severe impairment of the cone system. These results are consistent with the clinical characteristics of PCD.

At present, there are only a few reports on the SD-OCT characteristics of eyes with PCD. Mochizuki et al. and Baek et al. reported that the parafoveal retina was thinner, but the central macular area had normal thickness [5, 6]. The SD-OCT images in their patients also showed normal EZ and IZ throughout the macular region [5, 6]. In addition, Mochizuki et al. [5] also reported a thinning of the outer nuclear layer (ONL) in the parafoveal area. These findings suggest a degeneration of the parafoveal photoreceptors. The SD-OCT images from our patient showed a thinning of the parafoveal retina and attenuation of the parafoveal EZ and IZ. These findings are not completely consistent with earlier observations; however, our patient may have been at a more advanced stage of PCD compared to the patients reported earlier.

The results of the AO analyses were consistent with the results of OCT. AO analysis showed a marked decrease in the cone density at 450 µm from the foveal center in the patient. The oval area of residual cone photoreceptors in the AO image was similar to the shape of residual IZ in the *en face* OCT images. These findings suggest a degeneration of the parafoveal cone photoreceptors. Because cone labeling and cone counting were performed manually, the estimated cone density may not be an accurate number of healthy cones. However, we noted that the cone mosaic was severely disorganized in the patient compared to normal controls, especially at 450 µm from the foveal center. A comparison of the EZ and IZ in the OCT images and the AO images of our patient showed that the disorganization of the cones in the AO images was consistent with the disrupted EZ and IZ in the OCT images. Also, a well-organized cone mosaic in the normal controls corresponded with clearly distinguishable EZ and IZ in the SD-OCT images. Because both the IZ in the SD-OCT images and the AO images focused to the IZ level showed morphological feature of the same region, the AO and SD-OCT findings in the case were in good agreement.

Genetically, the patient’s parents were unaffected and non-consanguineous which suggests an autosomal recessive inheritance. Kondo et al. [3] presented two siblings with PCD, but the causative gene for the PCD was not determined. Quite similar B-scan and *en face* SD-OCT findings from each eyes of our patient suggested that the pathology was inherited rather than due to inflammation or trauma.

Our findings indicate that the PCD diagnosed by full-field ERGs and perimetry is due to a reduction in the density of parafoveal and peripheral cones. Because we have investigated only one patient, our study does not allow us to draw strong conclusions on why the degeneration is greater in the parafoveal cone photoreceptors with relative sparing of the central cone photoreceptors. To identify the molecular pathogenesis of PCD may clarify the mechanisms of foveal sparing of cone degeneration.
